# Antimicrobial Properties of Capsaicin: Available Data and Future Research Perspectives

**DOI:** 10.3390/nu15194097

**Published:** 2023-09-22

**Authors:** Aristodemos-Theodoros Periferakis, Argyrios Periferakis, Konstantinos Periferakis, Ana Caruntu, Ioana Anca Badarau, Ilinca Savulescu-Fiedler, Cristian Scheau, Constantin Caruntu

**Affiliations:** 1 Department of Physiology, The “Carol Davila” University of Medicine and Pharmacy, 050474 Bucharest, Romania; 2 Elkyda, Research & Education Centre of Charismatheia, 17675 Athens, Greece; 3 Akadimia of Ancient Greek and Traditional Chinese Medicine, 16675 Athens, Greece; 4 Pan-Hellenic Organization of Educational Programs (P.O.E.P), 17236 Athens, Greece; 5 Department of Oral and Maxillofacial Surgery, “Carol Davila” Central Military Emergency Hospital, 010825 Bucharest, Romania; 6 Department of Oral and Maxillofacial Surgery, Faculty of Dental Medicine, “Titu Maiorescu” University, 031593 Bucharest, Romania; 7 Department of Internal Medicine and Cardiology, Coltea Clinical Hospital, 030167 Bucharest, Romania; 8 Department of Internal Medicine, The “Carol Davila” University of Medicine and Pharmacy, 050474 Bucharest, Romania; 9 Department of Radiology and Medical Imaging, “Foisor” Clinical Hospital of Orthopaedics, Traumatology and Osteoarticular TB, 021382 Bucharest, Romania; 10 Department of Dermatology, ‘Prof. N.C. Paulescu’ National Institute of Diabetes, Nutrition and Metabolic Diseases, 011233 Bucharest, Romania

**Keywords:** capsaicin, antibacterial actions, antifungal actions, antiparasitic actions, antiviral actions

## Abstract

Capsaicin is a phytochemical derived from plants of the genus Capsicum and subject of intensive phytochemical research due to its numerous physiological and therapeutical effects, including its important antimicrobial properties. Depending on the concentration and the strain of the bacterium, capsaicin can exert either bacteriostatic or even bactericidal effects against a wide range of both Gram-positive and Gram-negative bacteria, while in certain cases it can reduce their pathogenicity by a variety of mechanisms such as mitigating the release of toxins or inhibiting biofilm formation. Likewise, capsaicin has been shown to be effective against fungal pathogens, particularly *Candida* spp., where it once again interferes with biofilm formation. The parasites *Toxoplasma gondi* and *Trypanosoma cruzi* have been found to be susceptible to the action of this compound too while there are also viruses whose invasiveness is significantly dampened by it. Among the most encouraging findings are the prospects for future development, especially using new formulations and drug delivery mechanisms. Finally, the influence of capsaicin in somatostatin and substance P secretion and action, offers an interesting array of possibilities given that these physiologically secreted compounds modulate inflammation and immune response to a significant extent.

## 1. Introduction

Antimicrobial resistance is an emerging threat identified by the World Health Organization and represents a global concern due to newly-acquired resistance mechanisms in a multitude of pathogens [1]. Antimicrobial drugs misuse as well as clinical and non-clinical pathogen transmission have contributed to the development of antimicrobial resistance, therefore novel antimicrobials are actively researched to combat this menace [2].

In the last decades there has been an increasing interest in the development of new antimicrobial substances from plants, as evidenced by a multitude of research on the subject (e.g., [3,4,5,6,7,8,9,10,11,12,13,14,15,16]). This renewed interest is based on the long-standing medical practices of various traditional medical systems, where plants and their derived extracts have been reported to have a host of applications. One important such substance is capsaicin, a chemical compound derived from plants of the *Capsicum* species [17].

Capsaicin (8-methyl-*N*-vanillyl-6-nonenamide) is a nitrogen-containing substance belonging to the lipids group [18]. While capsaicin was a term originally used to refer to a host of compounds isolated from *C. oleoresin*, nowadays it is a substance-specific name, while the rest of the originally associated substances are called capsaicinoids [19].

When isolated in its pure form, capsaicin (C_18_H_27_NO_3_) is a solid, colourless, hydrophobic, highly volatile, and highly pungent substance [20], which will produce toxic fumes if heated to decomposition levels [21]. The naturally occurring form of capsaicin is its trans form [22]. The biosynthetic pathway of capsaicin was originally described in the 1960s [23,24,25,26]. A number of methods to artificially synthesize capsaicin have been described [27].

Based on its properties, capsaicin is already used in a number of applications, as a component of animal repellents [28,29,30], fragrances [31,32], pesticides [33,34], and also in veterinary medicine [35,36]. A number of medical uses were reported, most notably as a treatment for chronic pain [37,38,39,40] and pruritus [41,42,43,44]; other minor uses have also been described by various researchers [45,46,47,48,49,50,51,52]. It should be noted that capsaicin is also capable of inducing local inflammation [53], a process which can be objectively measured through modern imaging applications [54,55].

In this paper, we will present a thorough and representative view of the studies regarding the actions and effects of capsaicin against bacteria, fungi, protozoa, and viruses. For this review, we carried an exploratory search using the Pubmed database from the National Center for Biotechnology Information (NCBI) of the United States of America (available at https://pubmed.ncbi.nlm.nih.gov/; accessed on 25 August 2023) which includes over 36 million citations for the biomedical literature from MEDLINE as well as other scientific sources. We used “capsaicin”, “antimicrobial action”, “antibacterial”, “antifungal”, “antiviral”, and “antiparasitic” as keywords, and included all relevant papers on the topic. We further supplemented the search in other databases such Google Scholar and Scopus. For each pathogen, we presented its relative importance in a clear and comprehensive way, based on current clinical evidence. By also describing the molecular mechanisms underlying the antimicrobial effects of capsaicin, we hope to depict a complete picture of the current corpus of knowledge on the subject and point out promising future research perspectives including the need to develop and test new capsaicin formulations.

## 2. Antibacterial Properties of Capsaicin

In the last decades, the use of plant metabolites against bacteria has been on the foreground of phytomedical and microbiological research (e.g., [56,57,58,59,60,61]). Capsaicin in particular has been the focus of recent research as a potential solution against antibiotic resistance [62]. Apart from finding natural alternatives to antibiotics, this is important both for those patients in which some antibiotics may be toxic—typical examples include allergies [63,64,65], liver toxicity [66,67,68,69], and other side effects [70]—and also, and perhaps more significantly, due to the increasing antibiotic resistance [71,72,73,74,75,76,77]. The rapid increase in antibiotic resistance is mainly explained by the overuse of antibiotics [78,79,80] and the high adaptability of bacteria in general, which may survive, depending on the species, even in extreme environments such as hot springs (e.g., [81,82,83]). The research on the antibacterial properties of capsaicin is extensive (Table 1) and this leaves open many potential choices for new drug design. Minimum inhibitory concentration (MIC) represents the lowest concentration of an antibacterial agent which, under in vitro conditions, totally prevents the visible growth in the tested strain [84]. Assessing this value is relevant to ensure the effectiveness of the antibiotic substance while limiting its administration to prevent adverse effects.

### 2.1. Antibacterial Activity against Staphylococcus aureus

*Staphylococcus aureus* is a bacterium that can frequently colonize the human body [94]. However, it is also known to cause a variety of diseases ranging from food poisoning to infections of the skin, such as scalded skin syndrome [95], or in the most severe cases, pneumonia and bacterial endocarditis [96,97]. Its biochemical arsenal comprises many toxins such as its enterotoxin and its exfoliative toxins, which are responsible for the aforementioned food poisoning and skin infections, respectively, and its hemolysin, called α-toxin [98]. Regarding the enterotoxin of *S. aureus*, it should also be mentioned that it is a super-antigen [98]. The emergence of Methicillin-resistant *Staphylococcus aureus* strains, also known as MRSA, is an important factor of concern both in a medical setting and from an economic point of view [99,100]. There are several types of MRSA such as the healthcare-associated MRSA (HA-MRSA), the community-associated MRSA (CA-MRSA), and the livestock-acquired MRSA (LA-MRSA) [101].

Capsaicin has potent action against *S. aureus* [102]. Specifically, it has been shown to affect the cellular viability of staphylococcal cells, exhibiting partial to total bactericidal effects, depending on the tested variety and the dilution level [88]. The extract of Bhut Jolokia Red is particularly potent in this regard, exhibiting partial bactericidal action even at 1:16 dilution [88]. Other studies have concluded that the effects on the growth in *S. aureus* colonies are more pronounced in the variety Noga Bhut when compared with the variety Roja Bhut [86]. When tested on mice, evidence suggests that capsaicin can have a protective role in staphylococcal pneumonia, as it was found to suppress the production of α-toxin and alleviate the inflammatory reaction [103].

### 2.2. Antibacterial Activity against Group A Hemolytic Streptococci

Streptococcal infections are associated with several pathologies such as skin infections, pharyngitis, pneumonia [104,105], and a critical condition known as toxic shock syndrome (TSS) [98]. Furthermore, due to the nature of protein M, one of the bacterium’s major antigens, virulence is high and reinfection with different M strains is a possible occurrence [105,106,107]. Other important parts of this bacterium’s antigenic structure are its pyrogenic toxin and its erythrogenic toxin, which are classified as superantigens [108], while it should also be mentioned that the species belonging to the category of group A hemolytic streptococci, most notable of them being *S. pyogenes*, owe this trait of theirs to their hemolysin, streptolysin O [109]. Perhaps the most notable trait of Streptococcus are the so-called post-streptococcal diseases, a group of severe sequelae which includes glomerulonephritis, rheumatic fever, and rheumatic heart disease, brought about due to different types of hypersensitivity reaction [104,106]. Macrolides, for example erythromycin, are becoming less and less effective as resistant strains emerge, and this poses a problem in the treatment of streptococcal infections in patients who are allergic to β-lactam antibiotics to which the bacterium is still susceptible [87,106].

Capsaicin was found in vitro to affect the biofilm formation and epithelial cell adhesion of species belonging to Group A hemolytic streptococci, reducing their invasiveness, while also having bactericidal action [87]. Moreover, the hemolytic activity of these species was diminished by a notable amount [87]. Apart from *S. pyogenes*, *S. mutans* has also been found to be susceptible to capsaicin [102].

### 2.3. Antimicrobial Activity against Enterococcus Species

The most notable species of enterococci are *E. faecium* and *E. faecalis* [110]. In recent years, enterococci have become the source of a considerable number of nosocomial infections [111] which can be of high severity [112,113]. The emergence of vancomycin-resistant enterococci (VRE) is a source of concern which indicates that alternative treatment options should be looked into [114].

Research results indicate that capsaicin can be used to inhibit the growth in *E. faecalis,* although it should be mentioned that its MIC was among the higher ones during the tests conducted by Nascimento et al. [85]. This can be possibly attributed to the fact that this bacterium, like others inhibited by similar MIC values, such as *B. subtilis* and *P. aeruginosa*, utilize capsaicin as a nutrient for growth [115]. However, dihydrocapsaicin exhibited lower MIC values than capsaicin in the aforementioned study, while also presenting a selective bactericidal effect related to cellular wall characteristics [85].

### 2.4. Antimicrobial Activity against Bacillus Species

Bacteria of the Bacillus genus are aerobic [116] and have a characteristic spore-forming ability, becoming resistant to the action of disinfectants as well as unfavourable environmental conditions [117,118]. Most species of the Bacillus genus are not pathogenic, the most notable exceptions being *B. anthracis* and *B. cereus* which associate increased mortality [119].

*B. subtilis* is non-pathogenic but the study of the action of capsaicin against it could be beneficial in understanding the action mechanisms against the aforementioned pathogenic species. Evidence from different sources [85,120] suggests that capsaicin is capable of inhibiting the growth in *B. subtilis* though at a comparatively higher MIC than most other bacteria [85]. Conversely, the species *B. thuringiensis* did not seem to be nearly as susceptible to the action of capsaicin [102]. Although *B. thuringiensis* is not pathogenic for humans, this finding may be of relevance, given the use of *B. thuringiensis* as a biopesticide [121].

### 2.5. Antimicrobial Activity against Listeria monocytogenes

*Listeria monocytogenes* is a species of ubiquitous, intracellular bacteria responsible for foodborne pathologies, capable of causing severe complications such as meningoencephalitis, especially in risk groups like immunosuppressed individuals, as well as pregnant women and foetuses, where abortion and septic premature death/neonatal death can occur [122,123,124]. The bacterium owes its intracellular nature to a variety of factors, most notably its internalins, which enable it to enter the host cell, and its hemolysin, listeriolysin O, which enables it to escape intracellular vacuoles [123].

The response of *L. monocytogenes* when exposed to capsaicin varies in lab settings depending on the extract with some displaying bactericidal action and others displaying bacteriostatic action [88]. The extract of Bhut Jolokia Red seems to be among the most effective ones, exhibiting partial bactericidal action even at 1:16 dilution [88].

### 2.6. Antimicrobial Activity against Vibrio cholerae

*Vibrio cholerae* is the causative agent of one of the oldest diseases known to man, characterized by profuse diarrhoea, which can be commonly found in aquatic ecosystems [125,126]. There are several biotypes which have pathogenic properties, featuring a great number of virulence factors [127]. Resistant strains of *Vibrio cholerae* are causes of concern [128,129] necessitating the search for alternative methods of treatment. 

Capsaicin has been found to significantly reduce the release of cholera toxin by interfering with the transcription of txA, tcpA, and toxT genes while at the same time enhancing the transcription of the hns gene which, in turn, downregulates the transcription of the former genes [89]. It should be mentioned that these results were noted along different serogroups and biotypes of this bacterium [89]. This is an important finding since several among them are responsible for pandemics and, as previously mentioned, some have also developed resistance to conventional antimicrobial agents [130].

### 2.7. Antimicrobial Activity against Acinetobacter baumanii

*Acinetobacter baumannii* is implicated in pulmonary infections and septicaemia in immunocompromised patients [131]. Its ability to resist the action of antibiotics and survive in harsh environments [132,133,134] only serves to exacerbate its pathologic nature. Based on the research of Ozçelik et al. [90], capsaicin is effective against *A. baumannii* at a concentration of 64 μg/mL. Interestingly, the research of Guo et al. [135] showed a lack of direct action of capsaicin against colistin-resistant strains of this bacterium but noted potent synergistic action in the case of combinatory use of these substances in a dose-dependent manner, where colistin MIC was greatly reduced.

### 2.8. Antimicrobial Activity against Helicobacter pylori

*Helicobacter pylori* is a causative agent of gastric ulcer and gastric cancer that displays increased rates of resistance to previously effective antibiotics such as clarithromycin and, to a lesser extent, metronidazole [136]; it is often characterised by multi-drug resistance patterns [137]. 

Capsaicin has showed promising bacteriostatic results at in vitro testing [138]. Its effects are exerted at concentrations as low as 25 μG/mL with the best results being achieved at 50 μG/mL, indicating possible use as a treatment option [91]. The usefulness of capsaicin as a treatment option for *H. pylori* also extends to the fact that it has demonstrated the ability to downregulate the proinflammatory pathway NF-Kb when tested in vivo on mice [139], a finding corroborated by other researchers [140], thereby reducing the extent of the inflammatory response caused by the bacterium and the subsequent gastric damage [139].

### 2.9. Antimicrobial Activity against Salmonella typhimurium 

Salmonella is a common causative agent of foodborne pathologies, which is mainly found in poultry, eggs, and dairy products, that threatens public health worldwide [141,142]. This bacterium displays a great serovariability with over 2600 serotypes having been recorded [143] and with different strains exhibiting different degrees of antigenic variability [144]. There are several strains which are resistant to the action of antibiotics [145,146] and their number is increasing at an alarming rate [147] while, at the same time, the increased virulence of said strains leads to a higher mortality [148].

Capsaicin has been documented as having partial bactericidal effects against *Salmonella typhimurium* [88]. Pure capsaicin exhibits protein-inhibiting qualities while extract from the plant *Capsicum chinense* (*C. chinense*) seems to be even more potent in that regard at the same doses while also preventing infection of Vero cells [92]. Based on the aforementioned data, future studies will hopefully elaborate on the antibacterial actions of capsaicin against other strains of the pathogenic *Salmonella* spp.

### 2.10. Antibacterial Activity against Escherichia coli

*Escherichia coli* is a commensal bacterium found in the gastrointestinal tract which can cause opportunistic infections if it migrates to different locations or when the host becomes immune-suppressed [149]. There have been recorded different types of *E. coli*, namely the enteropathogenic *E. coli* (EPEC), the enterohemorrhagic *E. coli* (EHEC), the enterotoxigenic *E. coli* (ETEC), the enteroaggregative *E. coli* (EAEC), the enteroinvasive *E. coli* (EIEC), and the diffusely adherent *E. coli* (DAEC) [150]. The emergence of multi-drug resistant (MDR) *E. coli* poses a problem that must be addressed in alternative ways such as new antibacterial substances [151,152].

Capsaicin has been shown to have partial bactericidal effects on *Escherichia coli* O157:H7 [88]. The inhibitory nature of capsaicin against *E. coli* has been confirmed by another study [85] though other researchers’ findings indicate that capsaicin merely slows down its growth [120]. At any rate, the effects of capsaicin on *E. coli* colonies are more potent in the case of the variety Roja Bhut when compared with the variety Noga Bhut [86].

### 2.11. Antibacterial Activity against Klebsiella pneumoniae

*Klebsiella pneumoniae* is an opportunistic pathogen which infects people worldwide, accounting for one-third of the total Gram-negative bacterial infections [153], and poses a considerable threat particularly in the nosocomial environment [154] where it can cause severe pathologies [155]. Due to strains which are resistant to antibiotics, including even last-line antibiotics, alternative methods of treatment are a necessity [154,156].

There is research evidence which suggests that capsaicin can exert an inhibitory effect on the growth in K. pneumoniae [85]. The usefulness of capsaicin’s action against K. pneumoniae is backed up by similar findings of other researchers [157]. Similarly, a formulation containing honey/tripolyphosphate/chitosan nanofibers loaded with capsaicin and gold nanoparticles was found to have inhibitory action against several bacteria, one of which was a different strain of the bacterium in question called *Klebsiella rhinoscleromatis* [158].

### 2.12. Antimicrobial Activity against Proteus Species

*Proteus mirabilis* and *Proteus vulgaris* are the most notable species of their genus and they are associated with urinary tract infections, like cystitis and pyelonephritis, while there have also been recorded cases of asymptomatic bacteriuria in elderly patients as well as patients with type 2 diabetes [159,160,161]. Urinary stone formation [162] and catheter obstruction in catheterized patients [163] are also possible complications. The severity of the pathologies caused by the aforementioned bacteria can be very severe [162,164], especially given the fact that there is a risk of these urinary stones serving as a focal point for other bacterial infections [164]. Bacteria of the Proteus genus have grown resistant to the action of antibiotics [165,166] and there even exist some MDR Proteus strains [167,168,169,170]. *P. vulgaris* in particular has been implicated in resistant nosocomial infections [171].

Capsaicin is effective against *P. mirabilis* as shown by the research of Ozçelik et al. [90]. *P. vulgaris* on the other hand has shown resistance to the effects exerted by capsaicin in tandem with other substances, which was attributed to its ability to elongate itself and secrete a polysaccharide when in contact with surfaces [102].

### 2.13. Antimicrobial Activity against Pseudomonas Species

*Pseudomonas aeruginosa* is a bacterium which can cause localized as well as systemic infections which are at times mild but can reach life-threatening severity [172], and is also commonly associated with nosocomial infections [173]. Patients with cystic fibrosis and COPD in particular are a risk group for *P. aeruginosa* infections [174,175,176]. There are *P. aeruginosa* strains which are becoming resistant to the action of antibiotics, meaning that new treatment options must be sought [177,178].

Studies have demonstrated the inhibiting properties of capsaicin on the growth in *P. aeruginosa* colonies even though the MIC is relatively high when compared to that of the other bacterial species tested by Nascimento et al. [85]. Based on the research of Kushwaha et al. [93], capsaicin along with 6-gingerol was able to inhibit the production of rhamnolipids, phenazine, and quinolone among other compounds; this finding may be important in dealing with resistant strains during biofilm formation. Capsaicin has also been shown to slow down the growth in a different species, *Pseudomonas solanacearum* [120].

## 3. Capsaicin as an Antifungal Agent

Compared to bacteria, only a limited fraction of fungi are considered to be pathogenic to humans [179]. While the majority of common fungal infections are not life-threatening, some species, such as *Candida albicans* and *Aspergillus fumigatus,* can even cause life-threatening infections under specific circumstances. While not as prominent as other pathogens, still they represent a considerable threat [179] and the burden of disease is high at least in specific regions [180,181,182]. Resistance to antifungal drugs is also a matter of concern [183,184,185] as is their side effects in some cases [186,187,188].

The main focus of study for the antifungal effects of capsaicin has been two gena, Candida and Aspergillus (Table 2), which are among the most common human fungal pathogens.

### 3.1. Antifungal Activity against Candida spp.

*Candida* spp. are usually benign but under certain circumstances, particularly in the case of *Candida albicans*, they can cause several pathologies, for example in the oral cavity [192] with many women also contracting vaginal candidiasis [193,194]. However, they have also been implicated in systemic infections of life-threatening severity [195]; this is dependent on the presence of risk factors [196,197]. Lately, the problem has become most evident in the hospital setting [198,199].

There has been extensive research on the susceptibility of *Candida* spp. to capsaicin, with satisfactory results. Capsaicin exhibits notable inhibiting properties against *Candida albicans* [85]. This inhibition becomes evident at 1:4 and 1:8 dilutions, with the yeast cells being killed, while the potency of the researched extracts was highlighted by the fact that all of them achieved partial inhibition even at 1:16 and 1:32 dilutions [88]. Capsaicin has also been shown to reduce the mature biofilm of *C. albicans* by 70–89% [200]. It has been concluded that capsaicin exerts its effects on the yeast cells by preventing ergosterol biosynthesis in the cell wall, thereby altering their shape and compromising their integrity [200]. Other species of Candida, like *C. glabrata* and *C. tropicalis*, were even more susceptible than *C. albicans* with not only their biomass formation being inhibited, but likewise the former’s biofilm-formation capacity being greatly diminished [189]. Indicatively, the MIC of extracts from *Capsicum chinense* was 1500 μg/mL for *C. albicans* but only 187.5 μg/mL for *C. glabrata* [189]. The hemolysis produced was similarly reduced by a significant degree [189]. Another very important finding was the fact that the action of fluconazole against yeast cells is enhanced when combined with capsaicin, which means that there could be a viable way of preventing the development of resistance to the aforementioned drug [200].

### 3.2. Antifungal Activity against Aspergillus parasiticus

The species of Aspergillus which are most relevant from a medical point of view are *A. parasiticus* and *A. flavus*, as they produce aflatoxins, secondary metabolites with harmful effects on both humans and animals [201], most notably carcinogenesis, mutagenesis, and teratogenesis [202]. Due to climate change, these species of Aspergillus can now be found in the soil of many countries worldwide, including Europe [203,204]. Considering that the use of harmful synthetic insecticides is common practice for eliminating *A. parasiticus*, the need for more environment-friendly methods of eradication has emerged [205].

Nanoparticles containing capsaicin and chitosan were tested against *A. parasiticus*, and the results were promising in that the incorporation of capsaicin in chitosan-containing lipid nanoparticles maintained a good antifungal effect while reducing the toxicity of the formulation [205]. Research results indicate that capsaicin not only has an inhibitory effect on the growth in *A. parasiticus*, but it also interferes with the germination of its spores and reduces the production of the aflatoxins [190,191] by suppressing the expression of the relevant genes aflM, aflR, aflS, and especially aflD [190]. This means that capsaicin-based compounds could be a useful source of non-synthetic fungicides [191].

## 4. Capsaicin as an Antiparasitic Agent

While most parasites are a danger to human health in the areas in which they are endemic [206,207,208], a host of factors may facilitate their spreading [209,210]. Thus, it may be imagined that the burden of disease is potentially considerable [211,212,213] and may increase given the emerging resistance [214,215]; even more so, some antiparasitic drugs, like antimonials, can have significant side effects [216]. Currently, the focus regarding the antiparasitic properties of capsaicin is centred on two species (Table 3).

### 4.1. Antiparasitic Activity against Toxoplasma gondii

Toxoplasma is an obligate intracellular eukaryotic parasite with a great spread; in fact, it is estimated that it infects up to one-third of the world’s population [218,219]. Oftentimes, infections caused be this protozoon are mild or even asymptomatic [218,220]. Their severity can be life-threatening however in the case of immunocompromised patients and newborns, the latter due to congenital transmission [218,219,220]. Pathologies of the retina and of the central nervous system are the ones most commonly associated with this microorganism [218]. A particularly common problematic finding is the development of tissue cysts which can lead to relapses in case of rupturing when a robust immunity is not present [221,222]. The parasite has a complex life cycle with many forms, namely tachyzoites, bradyzoites, and sporozoites [221]. Regarding its vector, felines serve as its definitive host and the oocysts developed within them are quite resistant when exposed to environmental conditions [218]. Even though toxoplasma is highly antigenic [220], it has at its disposal many proteins that enable it to evade the defences of our immune system [218,219,220]. *T. gondii* in particular uses specialized secretory proteins which allow it to invade and replicate within the host cell by modifying some of the latter’s factors [219]. This is achieved by means of interfering with gene transcription and signalling pathways [220]. Pyrimethamine and trimethoprim are the main treatment options but, due to the fact that they cannot distinguish between the enzymes of the parasite and the host, they should be administered together with sulphonamides, most notably sulfadiazine [222]. The result is severe side effects and subsequently, lower compliance rates [222]. There are also mentions of drug-resistant *T. gondii* strains [223,224]. As such, searching for better alternatives is a medical necessity. 

Research results have shown that *T. gondii*-infected BeWo cells show inhibited proliferation when treated with non-toxic concentrations of capsaicin in twofold serial dilutions, with the half inhibitory concentration (IC50) against its tachyzoites being 42.12 μg/mL [189]. From a pharmacological point of view, combinatory use of pyrimethamine and sulfadiazine alongside capsaicin yielded much better results than both of the two drugs combined or capsaicin alone [189].

### 4.2. Antiparasitic Activity against Trypanosoma cruzi

The most notable species of Trypanosoma are *T. brucei*, *T. gambiense* and *T. rhodesiense*. The main associated pathology is sleeping sickness, a disease endemic to African countries [225]. Its vectors are the Glossina flies, without excluding transmission by other blood-sucking insects [226,227]. The drugs used for the treatment of this condition are of two categories; the blood–brain barrier-crossing drugs, indicatively melarsoprol, eflornithine and nifurtimox, and the non-blood–brain barrier-crossing drugs like pentamidine and suramin [228]. Resistant strains are not prevalent but nor are they unheard of [229]. There is also *T. cruzi*, which is the causative agent of Chagas disease, which also has a zoonotic transmission [229].

*T. cruzi* was found to be susceptible to the action of capsaicin, with its trypomastigotes being affected more than its epimastigotes [217]. Although the research did not manage to find the exact target of capsaicin, its efficacy is undeniable considering that it exerted its effects in nanomolar concentrations with a potency many times higher than benznidazole, the drug mainly used for treatment of Chagas disease [217]. Findings also suggested that a capsaicin-based treatment could have an oral administration, another upside as far as therapeutical considerations are concerned [217].

## 5. Capsaicin as an Antiviral Agent

There have been many studies in the last few years documenting the extensive burden of disease from common viral pathogens [230,231,232,233]. Given that for many of the most common viral pathogens, there is a pattern of increasing resistance to antiviral drugs and recombinant strains emergence [234,235,236,237,238,239], and many viruses are associated with severe persistent pathological features [240,241,242,243,244,245], the research on natural antiviral substances is ever more important. 

The emergence of the coronavirus disease 2019 (COVID-19) [246,247] highlighted that despite the important progress in antiviral medicines, there are still significant gaps in our antiviral arsenal. Given the existence of deadly viruses, which could be potential pandemic-inducing agents, like the Marburg [248,249] and Ebola [250,251] viruses, and the existence of other viruses where there is not any fully effective therapeutical scheme, like the rabies virus [252,253], the need for novel antiviral agents becomes ever more evident. Unfortunately, comparatively little research has been undertaken in the antiviral front, and at the moment, it can be certainly said that capsaicin is definitively effective against only a limited number of viral pathogens (Table 4).

### 5.1. Antiviral Activity against the Influenza Virus

The influenza virus is a highly contagious virus that affects mainly the respiratory system and is characterized by high antigenic variability due to the genetic alterations it often undergoes [256,257,258]. This is true especially for type A influenza which is prone to causing pandemics [258]. Influenza’s antigenic variability comprises the antigenic shift and the antigenic drift it exhibits, the former being the cause of the aforementioned pandemics [259] and the latter necessitating constant updating of the vaccines [257]. A variety of medication is used for treatment, most notably the protein M-inhibiting drugs amantadine and rimantadine, to which the virus is now greatly resistant [260], the cap-dependent endonuclease-inhibiting drugs like baloxavir and marboxil [261,262], which can also be used for prophylactic purposes [263], and neuraminidase-inhibiting drugs like oseltamivir [257]. However, the available treatment loses a considerable part of its efficacy if it is not administered within the first stages of the infection, with the first 24 h being the optimal time frame, and 48 h being the end of the period during which drug administration can be expected to reliably produce the desired outcome [258]. A very serious possible complication is Reye’s syndrome, a pathology with diverse symptomatology which occurs in children that are infected with a virus, the causes of which are not yet clear, but it is thought to be caused by consuming acetylsalicylic acid in the context of the viral infection [264].

A case study showed that capsaicin may be used effectively to affect the viral neuraminidase, which is integral in the cellular invasion process [254]. Interestingly, a type of capsaicin-sensitive neurons of the respiratory tract may be instrumental in combating influenza infections, after they have been activated by capsaicin [265], but this is still the subject of further research.

### 5.2. Antiviral Activity against the Lassa Virus

Lassa virus (LASV) is an endemic pathogen in West Africa responsible for causing a haemorrhagic fever by the same name [266]. The reservoir of the virus are the rodents *Mastomys nataliensis* [267,268]. The pathogen has been recently recorded outside of its endemic radius [268], a reason for concern among the healthcare professionals. The World Health Organization (WHO) has incentivized the development of a vaccine against Lassa [268], which should come as no surprise given its high morbidity and mortality [268] as well as the limited therapeutic options currently available [255].

Capsaicin has been found to be able to inhibit the entry of Lassa virus into permissible cells by blocking the LASV-GP mediated fusion and by binding directly to the LASV pseudovirions [255]. The inhibition of entry is made possible due to the fact that capsaicin affects the stable signal peptide-GP2 transmembrane region of the virus’ glycoprotein [255]. Despite Lassa’s great genetic diversity, capsaicin proved to be effective in comparable rates against multiple different strains [255].

## 6. Discussion

The rapid development of phytochemistry during the last decades offers new possibilities and opportunities in the fight against numerous different pathogens. The approaches of phytomedicine are based both in traditional medicine practises—there is extensive research on ethnobotany (e.g., [269,270,271,272,273,274])—and modern biochemical research. Capsaicin is just one of the numerous phytochemicals, such as kaempferol (e.g., [6,13,14]), quercetin [4,5,9], curcumin [275,276,277,278], coumarin (e.g., [3,7,279]) and allicin (e.g., [8,12,280]), which have been under research for quite some time for their antimicrobial properties. 

As presented in the paper, there is much evidence to suggest that a host of mechanisms exists offering promise that capsaicin, alone or in combination with other compounds, can be, albeit sometimes in high concentrations, effective in an antimicrobial role. As mentioned, most of the pathogens against which capsaicin has been tested, are not only dangerous, especially in immunocompromised hosts or in nosocomial settings, but exhibit an even increasing resistance to existing therapies.

It must be noted that capsaicin has a variety of proven health-related properties, namely analgesic, [281,282], antioxidant [283], anti-inflammatory [284,285], anti-cancer [286,287,288,289,290,291,292], cardio-protective [293], vasculomodulatory [294], and metabolic modulation [283,295,296] effects; more organ-specific effects have been also mentioned [297]. Apart from these actions, an extensive number of traditional applications of capsaicin have been reported from Central and South America [298,299] where the red chili peppers where first cultivated, and even India [300] and Eritrea [301]. Of particular note, the native chilli of India, ‘Bhoot Jolokia’ (Capsicum chinense Jacq.), is regarded as the hottest chilli in the world, and has a host of applications in Ayurveda, the traditional Indian medicine [302].

On another aspect, repeated administration of capsaicin was proved by numerous researchers to inhibit the production and/or action of substance P [38,282,303,304,305,306,307,308,309] at least when locally applied. This is important, because apart from the other actions of substance P [310,311,312,313], it is implicated in the negative effects of infection-associated inflammation in animal models [314,315] and in teeth [316]; a recent review, also examined the association between the defensive capacity of the respiratory system and substance P in the context of the COVID-19 infection [317]. Therefore, in theory, for a patient suffering from a pathogen susceptible to capsaicin, the compound could exert a dual action, both a direct inhibition of the pathogen and a lessening of the associated inflammation.

We would also like to note that capsaicin-sensitive neurons can release somatostatin [285,318] and it is already well known that somatostatin promotes anti-inflammatory and anti-nociceptive effects [319,320,321]; recently, capsaicin was shown to be able to induce the release of somatostatin from such nerve endings when applied in transdermal patches [322]. Somatostatin is also important in its immunomodulatory role in cases of infection-induced inflammation; despite some positive effects, the secretion of somatostatin seems to downregulate the immune system [323,324,325,326]. Therefore, we could hypothesise that a capsaicin-derived antimicrobial drug could, at least in sufficient concentration, both act directly against the pathogen itself and also modulate the immune response by promoting somatostatin activity. However, another aspect we should consider here is that somatostatin and its analogues are evaluated as anticancer agents [327,328,329,330,331,332] and this can have implications in the case of viral-induced cancers [333,334,335,336].

Another important aspect related to the antimicrobial properties of capsaicin are its applications in neuropathy. Peripheral neuropathy may arise due to various causes such as type 2 diabetes, metabolic disorders or due to a considerable number of infectious agents as discussed by Brizzi et al. [337] and De León et al. [338]. On the other hand, a number of antimicrobial agents may also cause peripheral neuropathy themselves [338]; as such it is important to consider the potential applications as an alternative in the treatment of infections where the antimicrobials have this particular complication. Furthermore, capsaicin can be used to treat neuropathic pain [339,340] and therefore we may propose that capsaicin preparations can be used to treat both infection-induced peripheral neuropathy and the original infection itself. The effectiveness of such a treatment regarding the neuropathy could be monitored by a number of blood tests as suggested by a recent study [341]. Metabolic and systemic imbalances may aggravate the condition and should be taken into consideration [342,343].

Nonetheless, some constraints must be mentioned at this point, regarding the pharmacokinetics and pharmacotoxicity of capsaicin. As a compound, it is liposoluble and can be consequently administered locally, orally, and systemically [344]. The gastrointestinal absorption of capsaicin varies from 50% to 90% via a passive mechanism [345]; its rapid metabolization yields a number of active metabolites [346,347]. The half-life of capsaicin differs based on its application, from 25 min in systemic administration [348] to about 24 h in local administration [349,350]. It has been proven that it is possible to prolong capsaicin release and thus effective half-life using a carbopol-based formulation [351] specifically for antimicrobial applications. The metabolic pathways of capsaicin, which are important from a pharmacological perspective have been the subject of extensive research [352,353,354]. The development could lead to various applications, including perioperative care of complicated surgical cases or long-term treatment of infections in areas where antibiotic permeation is reduced [355,356,357,358].

While capsaicin has current and potential clinical applications, its side effects, which can be severe depending on the dose and application site are not negligible; pulmonary [359,360], gastrointestinal [361,362], cardiovascular [363,364], and even CNS [365,366] adverse effects have been reported. The adverse effect of capsaicin when it comes into contact with the eyes are even more rapid and pronounced [367]. This is of even greater importance in patients with impaired judgment or vision deficit that may mistakenly apply or consume capsaicin outside of the recommended use [368,369,370]. Another possibility is the use of composite creams containing capsaicin, along with other materials like coconut oil, which preserve medicinal capsaicin properties, while having a lower cost [371], and perhaps less side effects; the beneficial synergistic effects of coconut oil and capsaicin have also been noted by Trbojević Ivić et al. [372]. The antimicrobial effects of coconut oil have already been studied by a number of researchers (e.g., [373,374,375,376,377]) and it has been proposed that it can be a realistic antibacterial solution, at least for local infections of mild-to-medium severity [378]; its combination with capsaicin may further enhance its potential.

The aforementioned data raise some serious issues, namely as to how capsaicin, in a medicinal formulation, can be used in an antimicrobial role, reaching, in the affected tissues, concentrations sufficient enough to be effective, but not as high as to cause unbearable or even life-threatening side effects. This issue is further compounded by the fact that capsaicin is toxic for children in lower doses compared to adults [379]; this is even more problematic when considering that typically, most pathogens are more dangerous for children than for immunocompetent adults. Finally, in potential overdose cases, there is no way to speed up the elimination of capsaicin. Rather, the only option is to treat the intoxication symptoms until it is excreted [380].

A possible answer to the limitations of systemic administration—local administration with patches, creams, and other methods being an easier matter—could be the use of nanoparticles to deliver capsaicin to its target tissues, in sufficient quantities. Nanoparticles are already considered as a potential effective carrier of antibiotics (e.g., [381,382,383]), while other nanoparticles themselves are being considered as theoretically useful antiviral agents [384,385,386]. The delivery of antifungal agents via nanoparticles is also possible [387,388,389], and lately nanoparticles are being considered in the research for antiparasitic drugs [390,391,392]. While most of this research deals with metal nanoparticles, lipid nanoparticles are also a potential solution as discussed by Date et al. [393]. Another potential option for external applications could be the lipid nanoparticles used in wound care [394]. Successful applications of capsaicin-laden nanoparticles in vitro have also been included in our paper [171,205].

Overall, capsaicin’s use as an antibacterial agent covers a wide spectrum of pathogens; namely bacteria, both Gram positive and Gram negative, belonging to Staphylococcus, Streptococcus, Bacillus, Listeria, Vibrio, Acinetobacter, Helicobacter, Salmonella, Escherichia, Klebsiella, Proteus, and Pseudomonas species, in addition to fungal species like Candida and Aspergillus, as well as the parasites Toxopasma and Trypanosoma and the Influenza and Lassa viruses. This is important because most of the aforementioned microorganisms are commonly encountered in clinical practice. Moreover, the rising resistance noted in some strains alongside the side effects associated with the usual antimicrobial agents highlights the need for auxiliary treatments.

## 7. Conclusions

We conclude that capsaicin has a number of demonstrable antibacterial, antifungal, antiparasitic, and antiviral actions, and at least in its antibacterial role it is also considered as a promising perspective. Although significant research has been performed on this subject, more experiments are required in order to determine the effects of capsaicin on a wider host of pathogens and to elucidate whether there are any undiscovered mechanisms of action. 

Experiments with the aim to determine the capsaicin-induced inflammation in the cases of infections with capsaicin-susceptible pathogens should also be performed, the end goal being the designing of dual-purpose drugs; those having both an antimicrobial and an anti-inflammatory potential. Indeed, in most of the cases examined in this paper, the antimicrobial concentrations are reasonably low, and even in cases when they are on the high end, capsaicin may still be useful due to a lack of resistance, especially in the case of bacteria. 

An overview of the antimicrobial actions of capsaicin may contribute to multiple fields, including chemistry, medicine, homeopathy, traditional medicine, as well as other areas. This may further serve as a starting point to additional research, especially in this era marked by the increase in antimicrobial resistance.

Future research perspectives on this topic may include the closer examination of antimicrobial actions of capsaicin reported by traditional medicine as well as the exploration of more efficient nanoparticle carriers for antimicrobial capsaicin formulations. However, in any case, the most important research effort should be directed towards the applications of capsaicin against pathogens which are resistant to currently available medications.

## Figures and Tables

**Table 1:** Antibacterial actions of capsaicin based on existing research.

Genus	Species	Extract from	MIC (μg/mL)	Year of Research	Reference
Gram Positive					
Staphylococcus	*S. aureus*	*Capsicum frutescens*	1.2	2014	[85]
		*Capsicum chinense*	not specified	2018	[86]
Streptococcus	*S. pyogenes*	*Capsicum* spp.	64–128	2015	[87]
Enterococcus	*E. faecalis*	*Capsicum frutescens*	25	2014	[85]
Bacillus	*B. subtillis*	*Capsicum frutescens*	25	2014	[85]
Listeria	*L. monocytogenes*	*Capsicum* spp.	not specified	2018	[88]
Gram Negative					
Vibrio	*V. cholerae*	*Capsicum* spp.	100	2010	[89]
Acinetobacter	*A. baumanii*	*Capsicum annuum* L.	64	2011	[90]
Helicobacter	*H. pylori*	*Capsicum* spp.	25	2005	[91]
Salmonella	*S. typhimurium*	*Capsicum chinense*	not specified	2022	[92]
Escherichia	*E. coli*	*Capsicum frutescens*	5	2014	[85]
		*Capsicum chinese*	not specified	2018	[86]
Klebsiella	*K. pneumoniae*	*Capsicum frutescens*	0.6	2014	[85]
Proteus	*P. mirabilis*	*Capsicum annuum* L.	32	2011	[90]
Pseudomonas	*P. aeruginosa*	*Capsicum frutescens*	10	2014	[85]
		*Capsicum* spp.	not specified	2018	[93]

**Table 2:** Antifungal actions of capsaicin based on existing research.

Genus	Species	Extract from	MIC (μg/mL)	Year of Research	Reference
Candida	*C. albicans*, *C. glabrata*, *C. tropicalis*	*Capsicum frutescens*	25 (MIC_100_)	2014	[85]
		*Capsicum chinense*	187.5–1500 (MIC_100_)	2022	[189]
Apsergillus	*A. parasiticus*	*Capsicum chinense*	68 (MIC_50_)	2020	[190]
			381 (MIC_50_)	2020	[191]

**Table 3:** Antiparasitic actions of capsaicin based on existing research.

Genus	Species	Extract from	IC_50_	Year of Research	Reference
Toxoplasma	*T. gondii*	*Capsicum chinense*	42.12 µg/mL	2022	[189]
Trypanosoma	*T. cruzi*	*Capsicum* spp.	0.26–6.26 µM	2020	[217]

**Table 4:** Antiviral actions of capsaicin based on existing research.

Family	Genus	Extract from	EC_50_ (μmol/L)	Year of Research	Reference
Orthomyxoviridae	Influenza	*Capsicum* spp.	n/a	2022	[254]
Arenaviridae	Lassa	*Capsicum* spp.	From 6.1 to over 30 (strain-dependent)	2020	[255]

EC_50_ = Half maximal effective concentration.

## Data Availability

Not applicable.
